# Supplementary material for: Knowledge, Behaviour and Attitudes Related to Sun Exposure in Sportspeople: A Systematic Review
Source: Int J Environ Res Public Health. 2022 Aug 17;19(16):10175. doi: 10.3390/ijerph191610175 (PMC9407896; doi:10.3390/ijerph191610175)
Supplement: Supplementary file 1 [file ijerph-19-10175-s001.zip › ijerph-1830353-supplementary.pdf]

Table S1. Prisma Check List

Section/topic	#	Checklist item	Reported on page #
**TITLE**			
Title	1	Identify the report as a systematic review, meta-analysis, or both.	1
**ABSTRACT**			
Structured summary	2	Provide a structured summary including, as applicable: background; objectives; data sources; study eligibility criteria, participants, and interventions; study appraisal and synthesis methods; results; limitations; conclusions and implications of key findings; systematic review registration number.	1
**INTRODUCTION**			
Rationale	3	Describe the rationale for the review in the context of what is already known.	1-2
Objectives	4	Provide an explicit statement of questions being addressed with reference to participants, interventions, comparisons, outcomes, and study design (PICOS).	2
**METHODS**			
Protocol and registration	5	Indicate if a review protocol exists, if and where it can be accessed (e.g., Web address), and, if available, provide registration information including registration number.	3
Eligibility criteria	6	Specify study characteristics (e.g., PICOS, length of follow-up) and report characteristics (e.g., years considered, language, publication status) used as criteria for eligibility, giving rationale.	3
Information sources	7	Describe all information sources (e.g., databases with dates of coverage, contact with study authors to identify additional studies) in the search and date last searched.	3
Search	8	Present full electronic search strategy for at least one database, including any limits used, such that it could be repeated.	3-4
Study selection	9	State the process for selecting studies (i.e., screening, eligibility, included in systematic review, and, if applicable, included in the meta-analysis).	3-4
Data collection process	10	Describe method of data extraction from reports (e.g., piloted forms, independently, in duplicate) and any processes for obtaining and confirming data from investigators.	3
Data items	11	List and define all variables for which data were sought (e.g., PICOS, funding sources) and any assumptions and simplifications made.	3
Risk of bias in individual studies	12	Describe methods used for assessing risk of bias of individual studies (including specification of whether this was done at the study or outcome level), and how this information is to be used in any data synthesis.	3
Summary measures	13	State the principal summary measures (e.g., risk ratio, difference in means).	3
Synthesis of results	14	Describe the methods of handling data and combining results of studies, if done, including measures of consistency (e.g., I^2) for each meta-analysis.	3

Table S2. Risks of bias of the studies included

	Selection bias		Performance bias	Attrition bias	Detection bias				Reporting bias	
	Did the study apply inclusion/exclusion criteria uniformly to all	Does the design or analysis control account	Did researchers rule out any impact from a	If attrition (overall or differential nonresp	Were the outcome assessors blinded	Were interventions/exposures assessed/defined using valid and reliable measures,	Were outcomes assessed/defined using valid and reliable	Were confounding variables assessed using	Were the potential outcomes prespec	Overall ROB rating

	comparison groups?	for important confounding and modifying variables through matching, stratification, multivariable analysis, or other approaches?	concurrent intervention or an unintended exposure that might bias results?	onse, dropout, loss to follow-up, or exclusion of participants) was a concern, were missing data handled appropriately (e.g., intention-to-treat analysis and imputation)?	to the intervention or exposure status of participants?	implemented consistently across all study participants?	measures, implemented consistently across all study participants?	valid and reliable measures, implemented consistently across all study participants?	ified by the researchers? Are all prespecified outcomes reported?	
Bakos et al.[20]	Yes	No	No	NA	NA	Yes	Yes	Yes	No	High
Buljan et al.[21]	Yes	NA	No	NA	NA	Yes	Yes	Yes	Yes	Moderate
Christoph et al.[22]	Yes	Yes	No	NA	NA	Yes	Yes	Yes	Yes	Moderate
Cohen et al. [23]	Yes	No	No	NA	NA	Yes	Yes	Yes	Yes	Moderate
De Castro-Maqueda et al.[25, 26]	Yes	No	No	NA	NA	Yes	Yes	Yes	Yes	Moderate

De Castro-Maqueda et al.[24]	Yes	No	No	NA	NA	Yes	Yes	Yes	Yes	Moderate
De Castro-Maqueda et al.[27]	Yes	No	No	NA	NA	Yes	Yes	Yes	No	Moderate
De Gálvez et al.[28]	Yes	No	No	NA	NA	Yes	Yes	Yes	No	High
Del Boz et al.[29]	Yes	No	No	NA	NA	Yes	Yes	Yes	Yes	Moderate
Doncel Molinero et al.[30]	Yes	No	No	NA	NA	Yes	Yes	Yes	Yes	Moderate
Duarte et al.[31]	Yes	No	No	NA	NA	Yes	Yes	Yes	Yes	Moderate
Fernández-Morano et al.[32]	Yes	No	No	NA	NA	Yes	Yes	Yes	Yes	Moderate
García-Malinis et al.[33]	Yes	Yes	No	NA	NA	Yes	Yes	Yes	Yes	Moderate
Gutiérrez-Manzanedo et al.[34]	Yes	No	No	NA	NA	Yes	Yes	Yes	Yes	Moderate
Hobbs et al.[35]	Yes	No	No	NA	NA	Yes	Yes	Yes	Yes	Moderate

Laffargue et al.[36]	Yes	No	No	NA	NA	Yes	Yes	Yes	No	High
McCarthy et al.[37]	Yes	No	No	NA	NA	Yes	Yes	Yes	Yes	Moderate
Petty et al.[38]	Yes	Yes	No	NA	NA	Yes	Yes	Yes	Yes	Moderate
Rivas-Ruiz et al.[39]	Yes	No	No	NA	NA	Yes	Yes	Yes	Yes	Moderate
Tenforde et al.[40]	Yes	No	No	NA	NA	Yes	Yes	Yes	Yes	Moderate
Walker et al.[41]	Yes	No	No	NA	NA	Yes	Yes	Yes	Yes	Moderate
Wysong et al.[42]	Yes	No	No	NA	NA	Yes	Yes	Yes	Yes	Moderate

NA: Not applicable; ROB, risk of bias
